# Parasites as negative regulators of cancer

**DOI:** 10.1042/BSR20180935

**Published:** 2018-10-23

**Authors:** Blanca E. Callejas, Diana Martínez-Saucedo, Luis I. Terrazas

**Affiliations:** 1 Unidad de Biomedicina, Facultad de Estudios Superiores Iztacala, Universidad Nacional Autónoma de México, Avenida de los Barrios 1, Los Reyes Iztacala, Tlalnepantla, Estado de México, 54090, México; 2 Laboratorio Nacional en Salud, Facultad de Estudios Superiores Iztacala, Universidad Nacional Autónoma de México, Avenida de los Barrios 1, Los Reyes Iztacala, Tlalnepantla, Estado de México, 54090, México

**Keywords:** helminth, inflammation, parasitic protozoa, tumor

## Abstract

Several environmental factors (chemical, physical, and biological) can cause the initiation, promotion, and progression of cancer. Regarding the biological factors, several studies have found that infections caused by some bacteria, viruses and protozoan, and helminth parasites are related to carcinogenesis. However, in recent years a different approach has been implemented on the antitumor impact of parasitic diseases caused by some protozoan and helminths, mainly because such infections may affect several hallmarks of cancer, but the involved mechanisms still remain unknown. The beneficial effects reported for some parasitic diseases on tumorigenesis range from the induction of apoptosis, activation of the immune response, avoiding metastasis and angiogenesis, inhibition of proliferative signals, to the regulation of inflammatory responses that promote cancer. In this work, we reviewed the available information regarding how parasitic infections may modulate cancer progression. Despite the fact that specific mechanisms of action on tumors are not yet totally clear, we consider that detailed studies of the antitumor action of these organisms and their products could lead to the discovery and use of new molecules from these biological agents that may work as adjuvant therapy in the treatment of various types of cancer.

## Introduction

Cancer is a set of diseases that are acquired during the development of the neoplastic cell [1,2] that shares characteristics such as sustaining proliferative signaling, evading growth suppressors, resisting cell death, enabling replicative immortality, inducing angiogenesis, activating invasion and metastasis, genome instability, inflammation, reprogramming of energy metabolism, and evading immune destruction. Carcinogenesis is multifactorial, where genes, microenvironment and lifestyle, among others, play a key role in the development of cancer. In addition, infectious diseases participate in modulating carcinogenesis. It has been largely known that viral infections are associated with several types of cancer (i.e. papilloma virus and cervical cancer), as well as bacterial infections (i.e. *Helicobacter pylori* and gastric cancer). Furthermore, parasitic diseases may play an important role in favoring carcinogenesis, for example, the *Schistosoma haematobium* infection is associated with cancer of the urinary bladder and the *Clonorchis sinensis* and *Opisthorchis viverrini* food-borne liver flukes are associated with cholangiocarcinoma of the liver; the latter have been classified as carcinogenic agents [3]. The role played by some parasitic diseases caused by protozoan or helminth parasites as inducers or promoters of cancer has been meticulously described recently [4]; however, its regulatory effect on tumorigenesis has received much less attention. In the present review, we have compiled a series of studies pointing out for a potential positive modulatory effect of several parasitic diseases on tumorigenesis by having an effect on several hallmarks of cancer.

### Different hallmarks of cancer are impacted by parasites and their products

**Figure 1:**
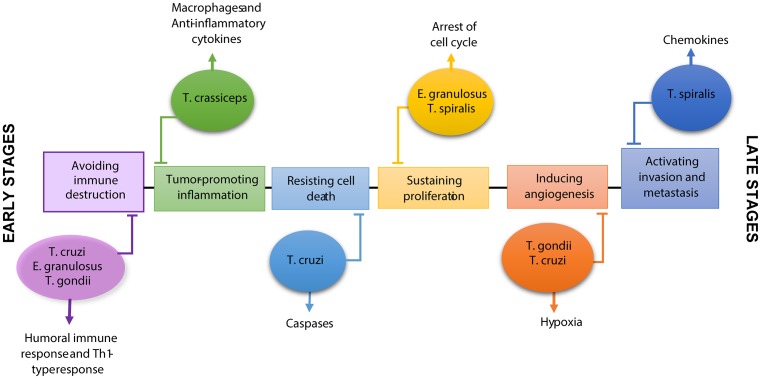
Parasites with therapeutic targeting of hallmark of cancer Protozoa such as *Toxoplasma gondii* and *Trypanosoma cruzi* have an antitumor effect on some cell types of cancer through the antiangiogenic capacity, reactivation of the immune response and induction of apoptosis. On the other hand, *Taenia crassiceps* is able to regulate the cancer-promoting inflammatory response. *Echinococcus granulosus* have different antitumor mechanisms such as reactivation of the immune response and antiproliferative effect on transformed cells, as well as *Trichinella spiralis* with regulating effect of invasion and metastasis and antiproliferative signals.

Hallmarks of cancer were defined some time ago and are well-known to include critical ‘factors’ that contribute to the immortality of neoplastic cells (Figure 1). Some of these hallmarks are affected by parasitic infections by modifying the immune response and, in consequence, altering the immune microenvironment of the tumor. For example, a dominant Th1-type response is displayed by the host during protozoan infections, whereas a dominant Th2-type response prevails in the host during helminth infections. Both of these responses will have an effect at least in two hallmarks of cancer, such as immune surveillance and inflammation. Thus, in this study we aimed at compiling several investigations that outline an important contribution of parasitic diseases and their products on modifying either positively or negatively some of the hallmarks of cancer. In this review, we focused on reports that suggest an inverse relationship between infections by some parasites and cancer, as well as its powerful therapeutic effect on modulating different hallmarks of cancer.

### Targeting hallmarks: avoiding immune destruction

The immune surveillance theory proposes that cells and tissues are constantly monitored by an immune system that is always alert; immune surveillance is responsible for detecting and eliminating preneoplastic and neoplastic cells. However, some cancer cells are able to evade the immune attack and its elimination [2]. The existence of immune surveillance has been demonstrated by the increase in the incidence of some types of cancer in immunocompromised patients, or in various animal models with elimination of one or more elements of the immune system. Due to the great importance of the immune system in the development of cancer, there has been attempts to develop immunotherapies directed against tumors with the aim of increasing the antitumor immune response and consequently the eradication of the neoplastic in progress. Among antitumor therapies, the use of helminths and protozoa for the reactivation of immune responses has been reported; such is the case of *Echinococcus granulosus, Toxoplasma gondii*, and *Trypanosoma cruzi*. It has been stated that different stages of the helminth parasite *E. granulosus* show antigenic similarity to mucin peptides and cancer cells, which is why several studies suggest the use of *E. granulosus* extracts as a potential inducer of antitumor activity for example, on increased activity of Natural Killer (NK) cells [5]. In addition, in an orthotopic model of colon cancer with the direct inoculation of the CT26 neoplastic cell line, prophylactic treatment based on the injection of hydatid fluid of *E. granulosus* generates antibodies capable of recognizing mortalin and creatine kinase M-type expressed on cancer cells, preventing in this way the establishment of carcinogenic cells and, therefore, tumor growth [6]. Other models described with the use of the extracts of this parasite will be mentioned later, since other antitumor mechanisms have been reported in addition to the activation of the immune response after exposure to some cancer cell lines.

Another parasite associated with an antitumor effect is *Trypanosoma cruzi*, which is a protozoan that causes Chagas disease. However, not all the information provided by this protozoan is negative, since some epidemiological studies report a lower incidence of colon cancer in patients infected with *T. cruzi* [7]. Several reports suggest that either the injection of *T. cruzi-*derived molecules or the infection with this parasite generated resistance to the development of some types of cancer. For example, in the orthotopic models of experimental breast and colon cancer, vaccination with epimastigotes of *T. cruzi* inhibited carcinogenesis through the activation of CD4^+^ and CD8^+^ cells, as well as by the increase of macrophages and dendritic cells, thus displaying greater NADPH oxidase activity. Also, antibodies directed against *T. cruzi* were able to specifically recognize human breast and colon cancer cell lines. Interestingly these antibodies also recognized 68% of tumor biopsies from breast and colon cancer patients [8]. Other reports suggest the use of the recombinant calreticulin of *T. cruzi* during the development of experimental breast adenocarcinoma, which reveals the presence of tumor cells to the immune system [9]. There are reports of other protozoa and their dual effect on tumorigenesis, which included epidemiological studies and *in vivo* models. *Toxoplasma gondii* is a protozoan parasite that induces strong polarization of Th1 responses in its host, with an increase in IFN-γ and IL-12 production, which is essential for resistance to this intracellular pathogen [10,11]. Due to the ability of *T. gondii* to modify the immune response of its host with this immunological profile, this parasite has been suggested as a potent inducer of antitumor responses. Therefore, the intratumoral administration of an attenuated strain of *T. gondii* in a melanoma model induced an immunogenic effect capable of stimulating the antitumor immune response, mediated by CD8^+^ T cells and NK cells, as well as increased expression of MHC-I and MHC-II molecules on antigen-presenting cells (APC) [12]. In addition, mice immunization with dendritic cells matured in the presence of *T. gondii*-derived profilin-like protein increased the activity of cytotoxic T cells and consequently, a decrease in the melanoma and fibrosarcoma tumor growth [13].

Therefore, some molecules constituting the surface of these parasites can induce the production of antibodies that recognize tumor cells due to the antigenic similarity between them, or they can serve as activators of cells involved in the process of cancer cells recognition. In addition, some infections by themselves can promote the antitumor response, such is the case of *T. gondii*. As mentioned above, it is characterized by the induction of IL-12 production, which in turn can stimulate NK cells and T cells to produce IFN-γ [14], regulating the expansion of CD8 T lymphocytes, as well as their cytotoxic capacity; therefore, promoting the activation of an antitumor immune response.

However, an epidemiological study conducted in a Chinese population suggests a general seroprevalence of *T. gondii* in patients with cancer, compared with those who did not suffer from this condition [15]. *T. gondii* DNA was detected in the transformed cells in two patients with primary intraocular B-cell lymphoma, but not in healthy tissue cells [16]. *T. gondii* tachyzoites were also detected in bronchoalveolar lavages in a patient with squamous carcinoma [17]. Another patient with anaplastic large cell lymphoma was diagnosed with active Toxoplasmosis [18], and similar data were observed in brain cancer. However, it is hypothesized concerning the latter that *T. gondii* potentially increases the risk of this neoplasia in humans through the inflammatory and antiapoptotic response generated by its encystment in the host brain [19]. Various reports regarding the effect of this protozoon on the development of certain types of cancer are still controversial. There is not enough information regarding a cause–effect relationship between *T. gondii* and carcinogenesis, so the correlation is poor and further information is needed to clarify them.

### Targeting hallmark: tumor promoting inflammation

At the beginning of the 19th century, Rudolf Virchow identified the accumulation of leukocyte infiltrate in samples of neoplastic tissue. This discovery marked the beginning of the possible association between inflammation and cancer [20]. Currently, several reports confirm a close relationship between inflammatory processes, proliferation, survival, and migration of cancer cells, as well as promotion of the release of agents that induce DNA damage [21,1]. In colitis-associated colorectal cancer (CAC), chronic inflammation plays a major role as an inducer and promoter of the neoplasm. At present, it is clearly recognized that patients with inflammatory bowel diseases as ulcerative colitis have an increased risk to develop CAC [22]. In accordance with Globocan 2012, colorectal cancer is the third most common cancer in men and the second in women worldwide [23]. To improve the study and provide new information regarding the advance and understanding of this increasing pathology, Tanaka et al. [24] developed a powerful and reproducible initiation-promotion model of colorectal cancer by using a chemical inducer of DNA damage, such as azoxymethane (AOM) followed by the exposure to an inflammatory chronic stimulus, such as Dextran Sodium Sulfate (DSS) that resembles colitis. Thus, the AOM/DSS model thoroughly resumes what happen during the process of colon carcinogenesis in humans [25]. This is a better model compared with orthotopic models that directly inject colon cancer cells subcutaneously into a tissue anatomically different from that where this cancer normally develops, given that all the process of DNA damage, inflammation, among others are lost. Taking advantage of the AOM/DSS model and with the knowledge provided by previous studies that state that the larval stage of *Taenia crassiceps* (larval stage that grows only in the peritoneal cavity) down-regulated the inflammation and improved the outcome of acute DSS-induced colitis [26], it was tested whether the previous extraintestinal infection caused by this helminth was able to inhibit the development of carcinogenesis in colon associated with inflammation. Interestingly, *T. crassiceps* preinfection reduced significantly (60%) the total number of tumors in the colon compared with uninfected mice, whereas 50% of the mice infected with this cestode did not develop tumors [27]. Such remarkable effect was associated with down-modulated recruitment of inflammatory monocytes and inhibition of local exacerbated inflammatory responses in the colon. In addition, this infection promoted alternative activated macrophages (M2) polarization and down-regulated the IL-17 production, as well as a reduction on the expression of several tumor markers such as β-catenin and COX-2 together with a Ki67 expression reduction, which is associated with cell proliferation. However, the specific antitumor mechanisms induced by the *T. crassiceps* infection during CAC development are still unknown, but some possible targets are suggested in Table 1. Currently, it has been observed that products released by the larval stage of *T. crassiceps* also regulate the development of CAC targeting on several intracellular pathways associated with tumorigenesis (personal observation). In contrast, an intestinal helminth infection caused by *Heligmosomoides polygyrus*, in the early stages of CAC promoted both inflammation and tumorigenesis in colon through the reduction of CD8^+^ effector T cells [28]. These contrasting results may arise from some differences in the parasitic models used, whereas *T. crassiceps* infection remains in the peritoneal cavity of their hosts and its chronic, *H. polygyrus* causes an intestinal infection with a limited life-span in this tissue. Moreover, in this last model, the DSS treatment could have damaged the epithelium in the small intestine and promoted an accelerated expulsion of the worm, thus avoiding a potential regulatory effect of *H. polygyrus* on tumorigenesis in the colon. Whereas *T. crassiceps* infection appears to positively modulate antitumor responses during CAC, there are reports with clear contrasting effects of other helminthic infections that seems to promote carcinogenesis such as those found in schistosomiasis and liver fluke, which were shown to be associated with bladder cancer and cholangiocarcinoma, respectively [29]. Therefore, more specific and more comprehensive studies are necessary to clarify the potential beneficial and/or harmful effects caused by helminthic diseases in different types of tumors.

**Table 1:** Parasites with putative antitumor activity

Parasite	Cancer	Mechanism of action	Reference
*Echinococcus granulosus*	Breast and colon cancer	Production of antibodies for the recognition of tumor cells	[5,6]
	Fibrosarcoma	Not clear	[37]
*Taenia crassiceps*	Colitis-associated colorectal cancer	Decrease recruitment of inflammatory monocytes and inflammation in colon	[27]
*Toxoplasma gondii*	Melanoma	Activation of CD8^+^, NK cells, and expression of MHC-I and MHC-II in APC	[12]
	Fibrosarcoma	Increase in the activity of cytotoxic T cells	[13]
	Melanoma and lung cancer	Suppression of neovascularization via induction of hypoxia and avascular necrosis	[31,32]
*Trichinella spiralis*	Melanoma	Reduction of lung metastasis through CXCL9, CXCL10, IL-4, CXCL1 and CXCL13	[30]
	Human hepatoma cell line (HT402) and human chronic myeloid leukemia cell line (K562)	Arrested of the cell cycle in G1 or S phase	[38,39]
*Trypanosoma cruzi*	Breast and colon cancer	Activation of CD4^+^ and CD8^+^ cells and production of antibodies against cancer cells	[8]
	Experimental breast adenocarcinoma	*Trypanosoma cruzi* calreticulin as a revealer of the presence of tumor cells in the immune system	[9]
	Mammary cancer	Inhibition of proliferation and migration of endothelial cells	[33]
	Melanoma	J18 recombinant protein induces apoptosis through caspase 3	[36]

The possible mechanisms of action of these parasites on blocking the development of different neoplasms has been organized with the purpose of summarizing the advances that have been made over the last years of research in immunotherapy with biological agents in cancer.

### Targeting hallmark: activating invasion and metastasis

The process of metastasis is a feature of carcinomas with a higher pathological degree of malignancy, in which the cancer cells acquire the ability to spread from the primary tumor to distant tissues. The first step is the invasion toward local tissues, then the intravasation of cancer cells to lymphatic tissue and nearby blood vessels, followed by the transport of these cells to distant tissues and their extravasation. The last step is to generate small nodules of cancer cells and gradually colonize new tissues. One of the many characteristics is that a cancer cell develops its ability to stimulate the production of chemokine ligands that promote its invasive behavior [2]. The effect of *Trichinella spiralis* on a melanoma model based on a subcutaneous injection of B16-F10 cells has been described within the immunotherapy with biological agents, where the previous oral infection with L1 larva of *T. spiralis* decreased tumor growth and its metastasis to the lungs by reducing the production of some chemokines, such as CXCL9, CXCL10, CXCL1, CXCL13, and IL-4 [30]. Interestingly, the increase of CXCL10 has also been associated with advanced human cancers such as malignant melanoma. In the present report, the group that was infected with *T. spiralis* and challenged with a melanoma cell line showed a greater reduction of CXCL10 production in comparison with the other group that was only infected with *T. spiralis*. This could imply that the immune profile generated by the infection could alter the response and change it into another antigenic stimulus, and such modification could have a regulatory effect on tumorigenesis, at least regarding this type of neoplasm.

### Targeting hallmark: inducing angiogenesis

The neovascularization associated with a tumor is generated by the process of angiogenesis, which is essential to supply nutrients and oxygen to the tumor cells for its neoplastic growth. It has been reported that protozoan infections are key in this process, thus during the acute phase of infection with *T. gondii*, there is an increase in the production of type II IFNs and cytokines that possess antiangiogenic properties. In an *in vivo* model of melanoma and in a Lewis lung cancer model, a *T. gondii* infection inhibited neoplastic growth through suppression of neovascularization via induction of hypoxia and avascular necrosis [31,32]. In addition, not only a liver infection may alter vascularization in a tumor, but also parasite-derived molecules may have an impact. For example, exposure to calreticulin derived from *T. cruzi*, in addition to promoting an antitumor immune response as mentioned above, may induce an antiangiogenic effect in breast tumors, both *in vitro* and *in vivo*, where *T. cruzi* calreticulin was able to inhibit the migration and proliferation of endothelial cells, possibly due to the internalization of this protein in the epithelial cells [33].

### Targeting hallmark: resisting cell death

Apoptosis is a physiological process of cell death triggered by intracellular signals or extracellular environment, which plays a critical role in the development and homeostasis of normal tissue. During the development of cancer, apoptosis functions as a barrier to contain the excessive proliferation of transformed cells. However, it can be attenuated in tumors with a higher degree of transformation and resistance to therapies [2]. Protozoa and helminths have been described as inducers of apoptosis, which is a survival mechanism, in cells of the immune system and epithelial cells [34,35]. This proapoptotic effect has also been tested in both *in vivo* and *in vitro* cancer models. The first signs of the antitumor effect of a parasite were observed in patients with chronic infections caused by *T. cruzi*. As it was previously mentioned, despite having the conditions for this tumorigenic process, epidemiological data indicate the absence of colon cancer in patients with chagasic megacolon [7]. Chronic inflammation caused by this pathology can lead to the development of mutations in epithelial cells or to the maintenance of these preneoplastic and neoplastic cells, which could suggest an important correlation between *T. cruzi* infection and a lack of development of colorectal cancer. Not only has the antitumor effect of *T. cruzi* infection been reported through the activation of the immune system, but also through a putative proapoptotic activity of the components of this protozoan. Some specific compounds from *T. cruzi* have shown a proapoptotic *in vitro* activity on several cell lines. For instance, the recombinant J18 protein based on gp82, a surface molecule of *T. cruzi*, induced apoptosis on melanoma cells without affecting the normal melanocytes. Furthermore, *in vivo* inoculation of recombinant J18 together with tumor cells induced tumors of smaller size. Other proapoptotic compounds detected in this parasite, such as Tc52, have been shown to down-modulate cell survival on different tumor cell lines, and this effect was associated with an increase in the activity of caspase 3 [36]. Therefore, such *T. cruzi*-derived molecules may reduce apoptosis-resistance in melanoma cell lines. This and other mechanisms of action of the *T. cruzi* components have not been fully described yet. For example, it is unknown whether these compounds are able to bind to some kind of receptor on the surface of the transformed cells or whether such compounds can directly cross the cell membrane and activate proapoptotic signals.

### Targeting hallmark: sustained proliferation signaling

Sustained chronic proliferation is a skill possessed by cancer cells through the deregulation of mitogenic signals. In an *in vitro* model of fibrosarcoma cells, treatment with protoscolices of hydatid cysts inhibited the proliferation of cancer cells [37]. Also, the crude extract of *Trichinella spiralis* inhibited the cell proliferation through the arrest of the cell cycle in the G1 or S phase of the human chronic myeloid leukemia cell line K562 and the hepatoma cell line H7402 [38,39]. The involved mechanisms are not clear yet, although during the formation of the *T. spiralis* cysts the protein p53 is expressed [40], which has the function of regulating the cell cycle, which may lead to the inhibition of tumor growth.

### Concluding remarks and future directions

In this review, we have gathered information about the different antitumor mechanisms triggered by some helminth and protozoan parasites, together they may target ∼50% of the hallmarks of cancer. In spite of having parasites that are classified as inducers and promoters of some neoplasms, others are reported as negative regulators of cancer. As described above, parasites can interfere in the growth and proliferation of a variety of transformed cell lines *in vitro*, but also, and more importantly, parasites and their products can modulate cancer development *in vivo* from melanoma to colon cancer. However, the mechanisms of action triggered by the parasites and some of their products involved in modulating cancer development are diverse and even not yet fully described. Their variability of antitumor response depends on several factors: the type of cancer and even the stage of transformation in which it is found, as well as the immune response generated by the host against the infection in progress, thus not all parasites and their molecules may have the same effect on carcinogenesis. Whereas some of them can activate the immune response in a by-standing way, such as unspecific activation of immune cells or inducing cytokines or reducing chemokines, other parasites can affect the cell cycle and stop cell proliferation in transformed cells (cell arrest) and generate a chance for the action of toxic drugs, thus promoting cancer elimination.

It is important to mention that the infections caused by helminths and protozoa to eliminate cancer could result in an impractical idea, given that such intervention may cause some harm to the host or unexpected infections by using alive parasites. However, this inconvenience may be outweighed by the identification of the products they secrete, as well as the molecules associated with their composition, with a direct effect on cancer cells or on the tumor microenvironment. This may be the key to create new antitumor treatments. The use of parasitic infections to modulate or impact positively on different inflammatory diseases and cancer should not be done with the aim of substituting the drugs in current use, but as a probe of concept for the potential use of their derivative molecules. We do not propose replacing the drug treatment with parasites or their molecules, but we rather propose to conduct more comprehensive studies on such potential molecules as regulators on cancer. Therefore, parasite biological factors may be used in a future as adjuvants to improve the effect of the current drugs. Although several *in vitro* studies have demonstrated that some molecules derived from parasites can induce apoptosis, only one study has been linked to the specific modulation of caspase 3 [36] and the remaining experiments have not defined the putative targets of such parasite molecules. Thus, it is mandatory to define more specific pathways affected by such treatments. For example, a common *in vitro* report states that exposure of cell lines or lymphoid cells to some parasitic extracts or semipurified molecules supports the idea of inhibition of cell proliferation [41]; therefore, it is essential to analyze the phases of the cell cycle that are being affected, such as the progression or arrest between the cell cycle G1, S, G2 and M phase, as well as to analyze the involved cyclins that may be down or up-regulated. Moreover, at least three different helminth infections have demonstrated to modulate the JAK–STAT signaling pathway [42] and to decrease, under certain circumstances, the NF-kB activity [43,44]. It is worth to note that such intracellular signaling pathways are consistently found altered in different types of cancer cells [45]. Thus, more detailed studies on the effects of helminths/protozoa and their products on these signaling pathways are crucial. In line with this idea, it has been reported that some helminth infections contribute to the overexpression of SOCS 1 and SOCS 3 [46,47], which blocks the JAK-STAT signaling, whereas other parasitic diseases have been found to inhibit STAT1 activation in response to IFN-γ [46]. Even such parasite-derived molecules may serve as ‘distractors’ to avoid the recruitment of harmful inflammatory cells in the tumor microenvironment. Additionally, a key research that must be conducted in the next few years is the search for putative receptors for parasitic-derived molecules in the immune cells or in the affected tissue, such as epithelial cells, as well as the signaling pathways that they may turn on or off in such cells.

Thus, the use of molecules derived from biological agents together with chemo and immunotherapy could be another key element to promote treatments directed against cancer. This is why, it is essential to have a deeper understanding regarding their potential antitumor activity.

## Abbreviations

AOM: azoxymethane
APC: antigen-presenting cells
CAC: colitis-associated colorectal cancer
DSS: Dextran Sodium Sulfate
